# Increasing Rates of Herpes Zoster Ophthalmicus and the COVID-19 Pandemic

**DOI:** 10.21203/rs.3.rs-2891711/v1

**Published:** 2023-05-09

**Authors:** Alexander J. Snyder, Hazem M. Mousa, Matias Soifer, Alessandro A Jammal, Sahil Aggarwal, Victor L. Perez

**Affiliations:** Duke University; Duke University; Duke University; Duke University; Duke Eye Center; Duke Eye Center

**Keywords:** Herpes Zoster, Herpes Zoster Ophthalmicus, VZV, COVID-19, Epidemiology

## Abstract

**Purpose:**

This epidemiologic study evaluates the variance in incidence of Herpes Zoster (HZ) and Herpes Zoster Ophthalmicus (HZO) within a single healthcare system with an aim to analyze their relationship to the COVID-19 pandemic.

**Methods:**

All patients attending the Duke University Health System (DUHS) from January 1, 2018, to December 31, 2021, were included. General and COVID-related trends of HZO and HZ were analyzed based on new ICD-9 or ICD-10 diagnosis codes, compared with the total number of patients seen at DUHS during this period, and the number of reported COVID-19 cases in North Carolina obtained using the CDC data tracker.

**Results:**

This study included 16,287 cases of HZ of whom 1,294 (7.94%) presented with HZO. The overall incidence of HZO showed an average yearly increase of 5.6%, however HZ incidence decreased by 5.3% per year. When comparing incidence rates of HZO in the 12-months before and after the COVID-19 pandemic onset in the United States (March 2020), the average incidence from March 2020 to February 2021 was 27.6 ± 11.6 compared to 18.0 ± 2.7 from March 2019 to February 2020 (p = 0.01). Moreover, 10/12 (83.3%) of the months had a higher incidence rate of HZO in the post-COVID onset year compared to their corresponding month in the pre-COVID year.

**Conclusion:**

The results show HZO incidence may be increasing, despite an overall lower HZ incidence. This could suggest a distinct mechanism for HZO appearance. The COVID pandemic, directly or indirectly, may have accelerated the already increasing HZO incidence.

## 1. Introduction

Herpes Zoster (HZ) or shingles is a painful reactivation of the varicella-zoster virus (VZV) in the sensory ganglia which spreads along an affected dermatome and is associated with significant morbidity and decreased quality of life.[1] HZ is known to affect 10–30% of people in their lifetimes and is estimated to occur in up to 50% of individuals over 85 years old.[1, 2] When HZ affects the ophthalmic branch of the trigeminal nerve, it is known as Herpes Zoster Ophthalmicus (HZO).[3] HZO is estimated to comprise 7–15% of HZ cases and is a common condition treated by ophthalmology practices.[1, 4–9] HZO typically presents as a unilateral localized painful rash in the periocular skin and eyelids, which includes eye involvement in about 50% of cases.[4–6] Keratitis is the most common ophthalmic manifestation, although HZO can directly compromise all ophthalmic structures including the cornea, conjunctiva, sclera, uvea, retina, and optic nerve.[4, 10] Chronically, HZO can cause neurotrophic keratopathy, conjunctival cicatricial changes and post-herpetic neuralgia (PHN), which is the onset of debilitating persistent neuropathic pain after 90 days and can lead to depression and suicide.[4, 10, 11] In addition, it has been reported that 8 to 31% of patients will develop a recurrent or chronic course of HZO.[10, 12]

Despite the clinical relevance, economic burden, and vaccination efforts, an increasing incidence of HZ and HZO has been reported in the United States over the past three decades ending in 2018.[5, 13–17] While there are many theories to account for this, the nature of the epidemiological increase is still unresolved. Additionally, although there is greater data supporting the increase in HZ trends, the epidemiological rates of HZO have been underreported. A recent large retrospective cohort study from 1994 through 2018 estimated a statistically significant increase in HZO incidence of 3.6% per year, with the disease being more frequent in females and Caucasians.[5] Since 2018, there is scarce data on the incidence of HZO and its ophthalmic clinical presentations. Having reports of such trends would allow for improved identification and surveillance, particularly in light of most recent major global events such as the coronavirus disease 2019 (COVID-19) pandemic.

COVID-19 is an acute respiratory disease caused by the highly transmissible virus, severe acute respiratory syndrome coronavirus 2 (SARS-CoV-2), that emerged in late 2019, causing a pandemic.[18] To date, little research has been conducted to evaluate the incidence of HZ before and after the COVID-19 pandemic. A Brazilian report suggested an increase in HZ diagnosis in 2020, as compared to the three previous years from 2017 to 2019.[19] Another study which reviewed insurance claims within the United States found a 15% higher incidence of HZ in adults who had been diagnosed with COVID-19 compared with matched controls.[20] Importantly, to date there are no studies specifically evaluating HZO incidence amidst the COVID-19 pandemic.

The purpose of this analysis is to determine the variation in incidence of HZ and HZO since 2018 within a single healthcare system with a focus on its correlation to the COVID-19 pandemic. We hypothesize that HZO rates have been steadily increasing and this has been further amplified by the COVID-19 pandemic.

## 2. Methods

### 2.1. Study Characteristics

This is a hospital-based single-center epidemiology study of patients attending Duke University Health System (DUHS) consecutively from January 1, 2018, to December 31, 2021. The study was approved by the Duke University Institutional Review Board (Pro00109493) with a waiver of informed consent. It adhered to the tenets of the Declaration of Helsinki for human subject research and adhered to the Health Insurance Portability and Accountability Act.

All patients presenting to the DUHS with a new diagnosis (i.e., first documented occurrence) of either HZO or HZ within the study period were included in the analysis. Subjects were identified via the Duke Enterprise Data Unified Content Explorer (DEDUCE) tool using the International Classification of Disease (ICD) 9th and 10th revision codes. The search included codes for HZO (ICD-9 0532.X; ICD-10 B023.X) and HZ (ICD-9 053.XX; ICD-10 B02.XX). Additionally, the total number of subjects seen at the DUHS in clinical visits for any medical reason within the same period were used to evaluate trends in the incidence of HZO and HZ, accounting for the drop in the number of overall encounters due to restrictions related to the COVID-19 pandemic.

Data was collected separately for patients with HZO as well as the HZ parent group with emphasis on demographics and chronologic trends. Demographic data included age, sex, self-reported racial group (i.e. Caucasian, African-American, Asian, or other), and self-reported smoking status at the time of diagnosis of HZO or HZ. General and COVID-related chronologic trends were analyzed by collecting data on the number of new consults for HZO or HZ, the total number of patients seen at the DUHS, and the number of reported COVID-19 cases in North Carolina obtained using the Centers for Disease Control and Prevention (CDC) data tracker (available at https://covid.cdc.gov/covid-data-tracker/#datatracker-home, access date January 15, 2022).

### 2.2. Outcomes and Statistical Analysis

The main outcomes of this study were: (1) recognizing overall and age group-specific trends in the incidence rate of HZO and HZ across the study period; and (2) investigating the impact of COVID-19 on the incidence rate of HZO by recognizing changes in trends after the onset of the pandemic (estimated onset in March 2020).

HZO and HZ rates were reported as the number of cases per 100,000 DUHS patients evaluated. COVID-19 data were obtained using the CDC COVID data tracker. DUHS likely underreported the true burden of COVID-19 on this patient population given a positive test result could be documented outside the hospital setting. The CDC COVID Data tracker was felt to provide a more comprehensive and accurate assessment of the COVID disease burden in the community.

A summary of characteristics at presentation was created using descriptive statistics. Statistical analyses were performed using SPSS software (version 25.0; SPSS Inc, Chicago, IL) with a 2-tailed cutoff of P < 0.05 for statistical significance. Paired non-parametric t-tests were used to compare monthly incidence rates of the year before (March 2019 to February 2020) and after (March 2020 to February 2021) the outbreak of COVID-19 in North Carolina. A Spearman’s rank correlation coefficient was used to assess for correlation between COVID-19 numbers and incidence rates of HZO across the 24 months preceding and following the COVID-19 onset (February 2019 to February 2021).

## 3. Results

### 3.1. Overall Patient Demographics

This study included 16,287 cases of HZ of whom 1,294 (7.94%) presented with HZO at DUHS from January 1, 2018, to December 31, 2021 (Table 1).

For patients with HZO, the average age was 60.9 ± 17.1 years (range 0 to 93 years) at the time of occurrence, with 55.8% of patients greater than 60 years old at presentation. Most subjects with HZO (59.7%) were female and 73.0% were self-identified as White or Caucasian. The population of active smokers and former smokers were also noted at 7.8% and 29.4%, respectively (Table 1).

At the time of first occurrence of any type of HZ, the average age was 58.5 ± 17.1 years, ranging from 0 to 95. Of these patients, 49.1% were greater than 60 years old, 63.5% were female, and 70.8% were self-identified as White or Caucasian. The population of active smokers and former smokers were 7.6% and 29.3%, respectively (Table 1).

**Figure 1:**
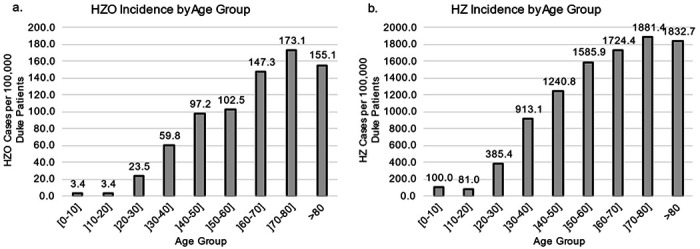
HZO and HZ incidence across age groups per 100,000 DUHS patients

Regarding age, the incidence of HZO increased at an average rate of 18.9 cases per 100,000 with increasing age decade group, reaching a peak of 173.1 HZO cases per 100,000 patients in the 70-to-80-year age bracket (Figure 1A). The incidence of HZ increased at an average rate of 216.6 per 100,000 DUHS patients with increasing age decade group, reaching a peak at 1881.4 cases per 100,000 DUHS patients in the 70-to-80-year age bracket, (Figure 1B).

### 3.2 General Trends with Time

**Figure 2:**
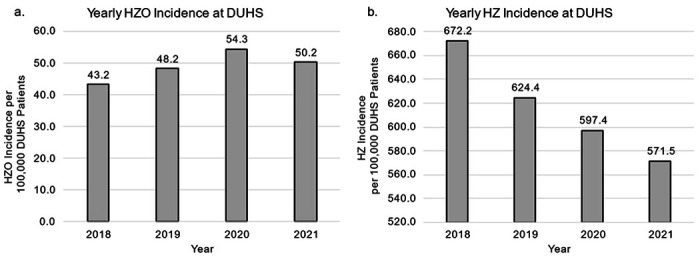
HZO and HZ incidence across time per 100,000 DUHS patients

Regarding changes over time, the incidence of HZO increased yearly during the study period from the baseline of 43.2 cases per 100,000 patients in 2018 up to 54.3 in 2020 with an average yearly increase of 2.3 cases per 100,000 patients or a 5.6% increase across the 4-year period (Figure 2A). In contrast, the incidence of HZ per 100,000 DUHS patients overall showed a steady decrease from 672.2 cases per 100,000 patients in 2018 to 571.5 in 2021, an overall yearly decrease of 33.5 cases per 100,000 DUHS patients or a 5.3% decrease between 2018 and 2021 (Figure 2B). Accordingly, the proportion of HZO cases specifically within the HZ group accounted for 6.7% in 2018 and 8.8% in 2021, which translated to an average yearly increase of 0.8%.

**Figure 3:**
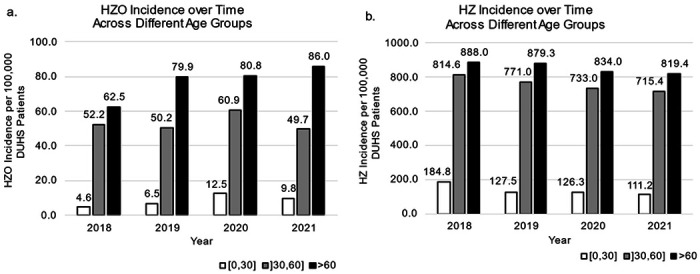
HZO and HZ incidence across time within each age group per 100,000 DUHS patients

Specifically, an increasing rate of HZO cases per 100,000 patients was noted for patients less than or equal to 30 years of age with an average yearly increase of 37%, and for those greater than 60 years of age with an average yearly increase of 12% between 2018 and 2021 (Figure 3A). The incidence of HZO per 100,000 patients within the ]30-60] year age group remained stable overall, but a spike was noted in the year 2020, reaching 61 cases per 100,000 patients (Figure 3A). In contrast, the incidence of HZ per 100,000 patients decreased by 14%, 4%, and 2% in the 30-year-old or less, ]30-60] year-old, and greater than 60-year-old groups respectively (Figure 3B).

In terms of changes in age group composition over the study period, the percentage of patients with HZO within the 30 and younger group increased from 2.9% in 2018 to 5.8% in 2021 with an average yearly increase of 0.96%. For HZ, the patients within the 30 and younger age group decreased from 7.4% in 2018 and stabilized at 5.8-6% between 2019 and 2021.

### 3.3. Trends since COVID-19 Pandemic Onset

**Figure 4:**
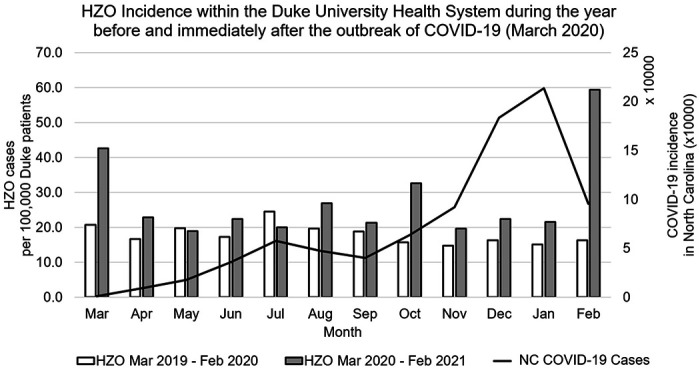
HZO Incidence per month in comparison to COVID-19 incidence in North Carolina for the year immediately before and after the COVID-19 outbreak

When comparing incidence rates of HZO in the 12-months before and after the outbreak of the COVID-19 pandemic in the United States (March 2020), the average incidence of HZO per 100,000 DUHS patients from March 2020 to February 2021 was 27.6 ± 11.6 compared to 18.0 ± 2.7 from March 2019 to February 2020 (p=0.01) (Figure 4). Moreover, 10/12 (83.3%) of the months had a higher incidence rate of HZO in the post-COVID year compared to their corresponding month in the pre-COVID year which was significant on a paired t-test (p=0.003). Analysis between March 2019 and February 2021 showed a significant and positive correlation between COVID-19 numbers and the monthly incidence of HZO per 100,000 patients (r= 0.59, p=0.002).

## 4. Discussion

This study observed the incidence rates of HZO and HZ within a single healthcare system over the 48-month period from January 1, 2018, through December 31, 2021, and analyzed general chronologic trends with focus on demographics and the impact of the COVID-19 pandemic on these rates.

We found that HZO represented 7.94% of all HZ cases, being more frequent in patients greater than 50 years old, Caucasian, and female. This rate and demographics are highly comparable to those reported using ICD-9 and ICD-10 code extraction by Berlinberg et al of 7.8% and Kong et al 7.9%, which validate our code extraction methodology.[5, 8]

With regards to timely trends, we noted that HZO increased yearly by 2.3 cases per 100,000 DUHS patients on average, corresponding to a 5.6% yearly increase. This corresponds well with the increasing rate reported in study by Kong et al from 1994 through 2018 of an increase at a rate of 1.1 cases per 100,000 person-years or 3.6% annually.[5] Conversely, the rate of HZ displayed a steady yearly decrease by 33.5 patients per 100,000 patients over the same period, subsequently resulting in an increasing HZO:HZ ratio over time.

The statistically significant increase in the ratio of HZO:HZ cases over the 4-year study may suggest a distinct mechanism for reactivation of the varicella virus within the trigeminal ganglion in comparison to more caudal locations with activation from dorsal root ganglia of the spinal cord. It is possible that the Zostavax ^®^ (Zoster Vaccine Live: ZVL; Merck & Co, Inc, Whitehouse Station, NJ) and Shingrix^®^ (Recombinant Zoster Vaccine: RZV; GlaxoSmithKline, Philadelphia, PA) vaccines are less effective at preventing outbreaks within the trigeminal ganglion and relatively more effective within more caudal locations. It is also possible that increasing rates of systemic diseases such as diabetes, obesity, and hypertension and poor access to care for these conditions have influenced HZO and HZ activation patterns. Further work could attempt to establish a distinct mechanism for reactivation of the varicella virus within the trigeminal ganglion in comparison to locations more caudal within the central nervous system.

Additional findings from this analysis were noted when sub-analyzing trends of HZO and HZ in different age groups. Across the study period, HZO rates had the highest increase in the bracket of patients under 30 years of age followed by the bracket of patients between 30–60 years of age with both groups comprising a higher proportion of the total HZO patients in 2021 compared to 2018. The increasing rate of HZO in younger patients was similarly highlighted in the study by Kong el al.[5] In contrast, the corresponding younger and middle age brackets demonstrated a decreasing rate of total HZ per year. These findings suggest the importance of considering an underlying HZO infection in patients with a suspicious presentation in these age groups despite it being nonclassical with regards to age or systemic findings.

Overall, HZO and HZ rates have been dynamic over time with several theories postulated to explain the trend of rising incidence of HZO. These include the increase in immunocompromised conditions and immunomodulatory therapies, immunosenescence within an aging population, health-seeking behaviors, access to healthcare, and the development of the varicella zoster vaccine in 1996 for pediatric patients which may be lowering wild-type exposure and therefore decreasing its immune boosting affects.[21–26] However, the ZVL and RZV zoster vaccines were introduced in the United States in 2006 and 2017 respectively to boost immunity against VZV reactivation. Recent research has indicated that the rate of HZ decreases in those who receive the ZVL or RZV vaccines.[24, 25, 27] Further studies will need to follow the multiple contributing factors to HZO rates but enhancing vaccination rates would likely be beneficial.

Another major outcome of the study was the investigation into correlations between HZO and HZ trends in relation to the COVID-19 pandemic that struck the United States in March of 2020. Our findings highlighted that the already increasing trend of HZO rates were further exaggerated after the onset of the pandemic with a positive correlation between reported monthly COVID-19 numbers and the corresponding monthly rate of HZO. The mechanism of association between the COVID-19 pandemic and HZ has not yet been fully elucidated. This study did not directly contrast patients with COVID-19 against matched controls. However, there are theories as to why HZO and HZ rates could be increasing within the COVID-19 pandemic. Wang et al found lower total lymphocyte, CD4 + T cell, CD8 + T cell, B cell, and Natural Killer cell counts in COVID-19 infected patients compared to healthy controls.[28] Xu et al. showed a significant decrease of T cell subsets during COVID-19 infection, which was similar to SARS infection but not common among other virus infections.[29] Studies prior to the COVID-19 pandemic had correlated decreased T cell function with HZ infection rates.[30, 31] This effective immunosuppression, specifically the decrease in cell-mediated immunity from T cell decline is a leading theory for increased HZ infections.[30, 31] Another theory may be that increased levels of stress within the pandemic have caused lower cell-mediated immunity, independent of COVID-19 infection, and are causing zoster rates to increase. Elevated levels of stress could explain the increased HZO rate at the onset of the COVID-19 pandemic in March 2020, despite low COVID-19 incidence within North Carolina at that time.

The limitations of this study include the data collection deriving from a single healthcare system within the southern United States, which may not be generalizable to disparate geographic locations. Additionally, the tertiary practice nature of the university facility may lead to a selection bias. Nonetheless, the health system data does include patients of all demographic groups and includes patients regardless of insurance status. Another limitation stems from coding practices, since some cases of HZO were likely billed with the general HZ diagnosis code, and the total number of HZO cases may be higher than reported. Given the limited data available on active COVID-19 infection, this analysis was not able to be completed within the patient population. Finally, it would have been beneficial to have herpes zoster and COVID-19 vaccination status data for stronger evaluation of associations. However, these are not often accessible within the health system database as many patients will receive vaccination through an outside pharmacy or care provider. Despite these limitations, we have shown that HZO rates are increasing, and COVID-19 may have accelerated it, despite decreasing HZ rates. Future multicentric reports with longer study periods and HZ vaccinations status are warranted to confirm these findings.

## 5. Conclusions

This study investigates the demographics and rates of HZO incidence in the USA since 2018 with a focus on the onset of the COVID-19 pandemic. We have observed that HZO incidence may be increasing, despite an overall lower HZ incidence. This could suggest a distinct mechanism for HZO appearance despite vaccination efforts, which should guide physicians towards early recognition of HZO. The COVID pandemic, directly or indirectly, may have accelerated the already increasing HZO incidence.

## Figures and Tables

**Table 1.** Demographics and clinical characteristics of subjects included in the study based on International Classification of Disease (ICD) for herpes zoster (HZ) and herpes zoster ophthalmicus (HZO).

Characteristic	HZO (n=1294 subjects)	HZ (n=16287 subjects)
Age, years	60.9 ± 17.1	58.5 ± 17.1

Sex, n (%)		
Female	772 (59.7%)	10349 (63.5%)

Race, n (%)		
Caucasian	944 (73.0%)	11525 (70.8%)
African American	213 (16.5%)	2958 (18.2%)
Asian	41 (3.2%)	565 (3.5%)
Other	14 (1.1%)	381 (2.3%)
Not reported	82 (6.3%)	858 (5.3%)

Smoking Status, n (%)		
Current Smoker	101 (7.8%)	1231 (7.6%)
Non-Smoker	769 (59.4%)	9826 (60.3%)
Former Smoker	380 (29.4%)	4769 (29.3%)
Not reported	44 (3.4%)	461 (2.8%)

## Abbreviations

HZ: Herpes Zoster
HZO: Herpes Zoster Ophthalmicus
VZV: Varicella Zoster Virus
PHN: post-herpetic neuralgia
COVID-19: coronavirus disease 2019
SARS-CoV-2: severe acute respiratory syndrome coronavirus 2
DUHS: Duke University Health System
ICD: International Classification of Disease
DEDUCE: Duke Enterprise Data Unified Content Explorer
ZVL: Zoster Vaccine Live
RZV: Recombinant Zoster Vaccine
